# SARS-CoV-2 y RT-PCR en pacientes asintomáticos: resultados de una cohorte de trabajadores del Aeropuerto Internacional El Dorado de Bogotá, 2020

**DOI:** 10.7705/biomedica.5802

**Published:** 2020-11-15

**Authors:** Jeadran Malagón-Rojas, Claudia Gómez-Rendón, Eliana L. Parra, Julia Almentero, Ruth Palma, Ronald López, Yesith Guillermo Toloza-Pérez, Vivian Rubio, Juan Felipe Bedoya, Fernando López-Díaz, Carlos Franco-Muñoz, Jhonnatan Reales-González, Marcela Mercado

**Affiliations:** 1 Doctorado en Salud Pública, Universidad El Bosque, Bogotá, D.C., Colombia Universidad El Bosque Universidad El Bosque BogotáD.C Colombia; 2 Dirección de Investigación en Salud Pública, Instituto Nacional de Salud, Bogotá, D.C., Colombia Instituto Nacional de Salud BogotáD.C Colombia

**Keywords:** infecciones por coronavirus, infecciones asintomáticas, síndrome de dificultad respiratoria del adulto, salud laboral, reacción en cadena de la polimerasa de transcriptasa inversa, Coronavirus infections, asymptomatic infections, respiratory distress syndrome, adult, occupational health, reverse transcriptase polymerase chain reaction

## Abstract

**Introducción.:**

La pandemia de COVID-19 ha ocasionado cerca de 25 millones de casos en el mundo. Se ha descrito que los pacientes asintomáticos pueden ser fuentes de transmisión. Sin embargo, es difícil detectarlos y no es claro su papel en la dinámica de transmisión del virus, lo que obstaculiza la implementación de estrategias para la prevención.

**Objetivo.:**

Describir el comportamiento de la infección asintomática por SARS-CoV-2 en una cohorte de trabajadores del Aeropuerto Internacional El Dorado "Luis Carlos Galán Sarmiento" de Bogotá, Colombia.

**Materiales y métodos.:**

Se diseñó una cohorte prospectiva de trabajadores del Aeropuerto El Dorado. El seguimiento se inició en junio de 2020 con una encuesta a cada trabajador para caracterizar sus condiciones de salud y trabajo. Cada 21 días se tomó una muestra de hisopado nasofaríngeo para detectar la presencia del SARS-CoV-2 mediante reacción en cadena de la polimerasa con transcriptasa inversa (RT-PCR). Se analizó el comportamiento del umbral del ciclo *(cycle threshold)* de los genes *ORFlab* y *N* según el día de seguimiento.

**Resultados.:**

En los primeros tres seguimientos de la cohorte se encontró una incidencia de la infección por SARS-CoV-2 del 16,51 %. La proporción de contactos positivos fue del 14,08 %. La mediana del umbral del ciclo fue de 33,53.

**Conclusión.:**

Se determinaron las características de la infección asintomática por el SARS-CoV-2 en una cohorte de trabajadores. La detección de infectados asintomáticos sigue siendo un reto para los sistemas de vigilancia epidemiológica.

## Abstract (en)

**Introduction:**

The 2019 coronavirus pandemic (COVID-19) has caused around 25 million cases worldwide. Asymptomatic patients have been described as potential sources of transmission. However, there are difficulties to detect them and to establish their role in the dynamics of virus transmission, which hinders the implementation of prevention strategies.

**Objective:**

To describe the behavior of asymptomatic SARS-CoV-2 virus infection in a cohort of workers at the El Dorado "Luis Carlos Galán Sarmiento" International Airport in Bogotá, Colombia.

**Materials and methods:**

A prospective cohort of 212 workers from the El Dorado airport was designed. The follow-up began in June, 2020. A survey was used to characterize health and work conditions. Every 21 day, a nasopharyngeal swab was taken to identify the presence of SARS-CoV-2 using RT-PCR. We analyzed the behavior of the cycle threshold *(ORFlab* and *N* genes) according to the day of follow-up.

**Results:**

In the first three follow-ups of the cohort, we found an incidence of SARS-CoV-2 infection of 16.51%. The proportion of positive contacts was 14.08%. The median threshold for cycle threshold was 33.53.

**Conclusion:**

We characterized the asymptomatic SARS-CoV-2 infection in a cohort of workers. The identification of asymptomatic infected persons continues to be a challenge for epidemiological surveillance systems.

La pandemia por coronavirus 2019 (COVID-19) ha generado múltiples y diversos desafíos a nivel mundial en todos los ámbitos laborales, no solo entre los trabajadores del área de la salud. En la semana epidemiológica 36, la Organización Mundial de la Salud (OMS) reportó más de 25 millones de casos en el mundo y la muerte de cerca de 800.000 personas por la enfermedad ^1^. El 80 % de los casos de COVID-19 son leves y asintomáticos ^2^^-^^4^.

La infección asintomática se define como la detección positiva del ácido nucleico del virus SARS-CoV-2 mediante reacción en cadena de la polimerasa con transcriptasa inversa (RT-PCR) en muestras de pacientes que no presentan signos clínicos ni radiológicos típicos de la COVID-19 ^5^. La incidencia de infecciones asintomáticas reportada oscila entre el 1,6 y el 56,5 % de los casos ^6^^,^^7^. Aunque la transmisión del virus por personas asintomáticas se ha reportado en varios lugares del mundo ^8^^,^^9^, el papel de este tipo de portadores en la diseminación de la infección aún no se ha aclarado ^10^.

La detección de potenciales portadores del SARS-CoV-2 supone un reto para los sistemas de vigilancia epidemiológica y, sobre todo, para la reactivación de la economía mundial afectada por las diferentes medidas adoptadas por los gobiernos para disminuir la transmisión del virus ^11^^,^^12^. Una de las estrategias implementadas en la vigilancia epidemiológica ha sido la búsqueda activa de casos mediante la toma de la temperatura y el registro de la presencia de síntomas ^13^, o las tamizaciones aleatorias en búsqueda de anticuerpos ^14^ o ARN viral ^15^.

Cada una de estas estrategias implica retos en términos de la capacidad de respuesta de los diferentes sectores involucrados en la detección y notificación de los casos. Una vigilancia activa que se centre en la captura temprana de casos con base en la identificación del virus puede ser más eficaz para cortar las cadenas de transmisión y reducir así el impacto económico del ausentismo y la pérdida productiva de las empresas a mediano plazo ^16^.

En esta comunicación se presentan los resultados del seguimiento en una cohorte de trabajadores con el propósito de analizar las características de las infecciones asintomáticas por SARS-CoV-2 y las implicaciones que tiene el uso de la RT-PCR como método de tamización en la población de estudio.

## Materiales y métodos

Se diseñó una cohorte prospectiva en un grupo de trabajadores del Aeropuerto Internacional El Dorado "Luis Carlos Galán Sarmiento" de Bogotá, Colombia. El estudio incluyó trabajadores sanos con contrato vigente y trabajo presencial en la terminal aérea.

La población objeto estaba constituida por 500 trabajadores. Se estimó un tamaño de muestra de 205 individuos (incidencia del 5 %; confianza del 95 %; porcentaje de pérdidas aceptable del 10 % y precisión del 2,3 %), usando como referencia las proyecciones sobre la incidencia de la COVID-19 del Ministerio de Salud y Protección Social ^17^.

La observación de los trabajadores se hizo entre el 1° de junio y el 31 de agosto de 2020. Al incorporarlos al estudio se les hacían la RT-PCR y la prueba serológica para confirmar que estuvieran sanos. Posteriormente, a cada trabajador incluido en la cohorte se le tomó una muestra de hisopado nasofaríngeo para la identificación del ARN viral cada 21 días. A los trabajadores que resultaron positivos se les hizo seguimiento en su domicilio cada siete días durante 21 días tomando muestras de hisopado nasofaríngeo y sangre total para la identificación de anticuerpos.

Las muestras nasofaríngeas fueron procesadas por el Instituto Nacional de Salud según lo señalado en el protocolo de Berlín para la RT-PCR ^18^, y se amplificaron los genes *ORFlab* y *N* para obtener los valores del umbral del ciclo *(cycle threshold,* Ct), cuya positividad se establece por debajo de los 40 ciclos.

Se incluyeron en el estudio los contactos familiares de los trabajadores con COVID-19. Se consideró como portadores asintomáticos de la infección a aquellos trabajadores con seguimiento durante 21 días que no desarrollaron ningún síntoma en dicho periodo.

### Análisis estadístico

Para las variables cuantitativas se estimaron los promedios, las medianas la desviación estándar y el rango intercuartílico (RI). Para las variables categóricas se establecieron frecuencias y porcentajes. Se estimó la incidencia acumulada en el periodo evaluado ^18^ y se utilizó la prueba de ji al cuadrado para establecer las diferencias entre hombres y mujeres en cuanto a los Ct. Además, se usó la prueba de Friedman para estimar las diferencias entre los grupos de seguimiento en las medias de los Ct. El nivel de significación estadística se estableció con un valor de p menor de 0,05. Se usó el paquete estadístico SPSS™, versión 23 (licencia del Instituto Nacional de Salud) y el programa R, versión 4.0.2 (2020-06-22) de acceso libre ^19^.

### Consideraciones éticas

El proyecto, el consentimiento informado por escrito de los participantes y los instrumentos de captura de la información fueron aprobados por el Comité de Ética y Técnico del Instituto Nacional de Salud mediante el acta 012 de 2020.

## Resultados

La cohorte prospectiva incluyó 212 trabajadores del Aeropuerto Internacional El Dorado "Luis Carlos Galán" de Bogotá. En el periodo de observación se tomaron 869 muestras de 212 trabajadores, correspondientes a cuatro seguimientos por trabajador como mínimo. El promedio de edad fue de 36,3 ± 8,2 años; el 73 % (n=155) de los trabajadores era de sexo masculino. Los estratos socioeconómicos predominantes fueron el dos y el tres (83,5 %, n=177); la muestra estaba conformada principalmente por mestizos y blancos (97,2 %, n=206). Se encontró que el 98 % (n=208) de los trabajadores encuestados usaba tapabocas desechables durante la jornada laboral y se lavaba las manos, por lo menos, una vez cada hora.

La incidencia de SARS-CoV-2 se estimó en 16,51 % (35/212). De este porcentaje, el 68,67 % (n=24) correspondió a casos asintomáticos y el 31,43 % (n=11) a casos sintomáticos.

En el seguimiento de las personas positivas la proporción de quienes siguieron siéndolo fue del 21,31 % (n=13) en el día 7, de 9,84 % (n=6) en el día 14 y de 6,56 % (n=4) en el día 21. En tres casos (dos hombres y una mujer) las pruebas de RT-PCR siguieron siendo positivas tras 28 días de seguimiento.

La proporción de contactos positivos entre los casos fue del 14,08 % (IC_95%_: 5,99-22,18 %; 10/71). El promedio de contactos fue de dos personas (IC_95%_: 2,27-2,65).

La mediana del umbral de Ct del gen *ORFlab* fue de 33,53 (Rl: 29,14 -36,61); la del gen *N* fue de 33,61 (Rl: 29,38 - 36,81) y la del Ct de control fue de 27,92 (Rl: 25,17 - 31,35).

**Figura 1:**
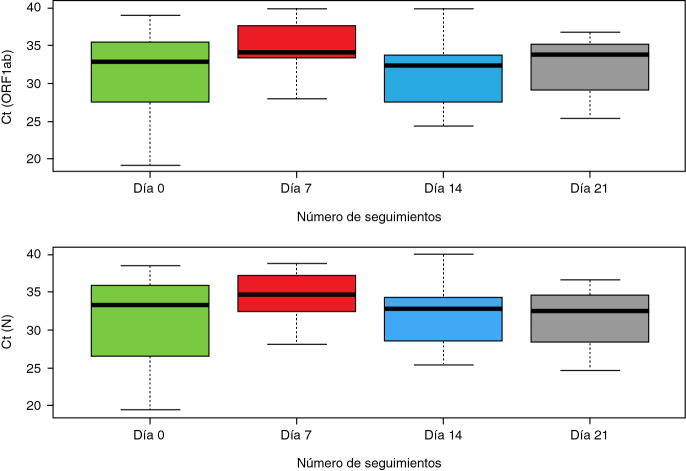
Distribución de los umbrales del ciclo *(cycle threshold,* Ct) según el día de seguimiento (n=61 muestras positivas)

En el análisis del Ct de cada gen en el momento del estudio (primera muestra positiva *Vs.* muestra positiva de seguimiento), se observó que la media tendía a ser mayor en la primera toma y a aumentar en las posteriores (figura 1). La prueba de Friedman evidenció diferencias significativas entre los días de seguimiento en las medianas del CT de los genes *ORFlab* y *N* (c^2^=60.000, p=0,000 y c^2^=59.000; p=0,000, respectivamente). No hubo diferencias significativas en el Ct entre hombres y mujeres (p=0,44) ni entre grupos de edad (p=0,51).

## Discusión

Se encontró una incidencia de la infección por SARS-CoV-2 del 16,51 % en el periodo de observación. De este porcentaje, el 68,67 % correspondió a portadores asintomáticos de SARS-CoV-2. El número de contactos cercanos de los casos asintomáticos osciló entre 1 y 5, cifra que difiere de la establecida en los ejercicios de modelado matemático diseñados en el primer semestre de la pandemia, en los que se estimaba el número de contactos cercanos entre 10 y 36 ^20^, y que se explicaría por la implementación de las medidas de distanciamiento social que habrían reducido el número de contactos en la vida diaria. Este hallazgo concuerda con lo reportado por Afzal, et *al.,* en cuyo estudio se determinó que el promedio de contactos por caso de COVID-19 era de siete personas ^21^.

Un resultado importante de este estudio fue que el 50 % de los positivos tuvieron valores de Ct por encima de 33 y la proporción de contactos cercanos infectados fue relativamente baja (11,86 %). Esto respalda dos planteamientos: primero, que la transmisión de la infección por asintomáticos no es tan eficiente como la de los pacientes sintomáticos y, segundo, que al tener Ct altos, la carga viral es baja y puede no ser suficiente para desarrollar la COVID-19 o para infectar a los contactos cercanos.

En este sentido, en una revisión de la literatura sobre el problema de la infección asintomática por SARS-CoV-2, se plantea que es apresurado centrar las esperanzas en la inmunidad colectiva producto de la transmisión del virus por contacto con pacientes asintomáticos dadas las bajas tasas de seroconversión de los portadores asintomáticos ^22^.

Sin embargo, estos resultados difieren de otros reportados por varios autores en torno al papel de las personas asintomáticas en la transmisión de la enfermedad. Ye, *et al.,* por ejemplo, señalaron que la transmisión del virus puede tener lugar en contextos de contacto estrecho, como las viviendas ^23^, en tanto que en su estudio sobre pacientes asintomáticos y contaminación de superficies, Wei, *et al.* encontraron que las personas asintomáticas con SARS-CoV-2 contaminaron su entorno y, por lo tanto, pusieron en riesgo a otras personas ^24^.

Aunque el presente estudio no incluyó un grupo de comparación, la transmisión fue baja, en parte porque la población analizada seguía las recomendaciones de protección. La baja transmisión entre los contactos podría explicarse por el cumplimiento reportado del uso de tapabocas y del lavado de manos (98 %). Desde el cierre del aeropuerto el pasado 22 de marzo se ha implementado en varios frentes de trabajo la instalación de máquinas dispensadoras de elementos de protección personal (tapabocas desechables N95 y caretas de protección), así como lavamanos portátiles y dispensadores de gel para higienizar, puntos de control de la temperatura y demarcación de áreas para limitar aglomeraciones, además de la capacitación de los trabajadores sobre la prevención de la transmisión del virus ^25^. El uso de estos elementos ha permitido la disminución del riesgo de infección en diferentes entornos ^26^. Llama la atención el comportamiento de los Ct a lo largo de los seguimientos. En el análisis estadístico se observaron diferencias significativas en las medianas de los grupos de días de seguimiento para los dos genes evaluados (mediana del Ct del *ORF1ab:* 33,53, RI: 29,14 - 36,61; mediana del Ct del N: 33,61, RI: 29,38 - 36,81). Estos hallazgos son similares a los reportados por Singanaya, *et al.*^27^ quienes reportaron el seguimiento de 324 casos de COVID-19 en Inglaterra durante el primer semestre de 2020 y observaron que la mediana de los Ct en pacientes asintomáticos era de 31,23 (RI: 28,21 - 32,97).

Un resultado interesante tiene que ver con el hecho de que los Ct de las personas asintomáticas se dan tardíamente y, aunque no hubo diferencias significativas importantes entre los grupos, es necesario resaltar que las medianas de los Ct fueron superiores a 33.

En la actualidad, el punto de corte para determinar que una prueba es positiva está establecido en un Ct entre 38 y 40 ^28^. Sin embargo, varios autores han propuesto replantear este punto de corte para la prueba diagnóstica de SARS-CoV-2. En un informe publicado por los *Centers for Disease Control and Prevention* (CDC) de los Estados Unidos se plantea la modificación del punto de corte del Ct para definir la recuperación en pacientes sintomáticos y se propone que sea de 33 ^29^. Por su parte, Binnicker, *et al.* encontraron en su estudio que cuando los Ct eran superiores a 34 la probabilidad de cultivar el virus decrecía significativamente y, por lo tanto, su capacidad de infectar a otros ^30^.

A la luz de la evidencia actual, la tecnología de cultivo viral disponible y la posibilidad de explotar el carácter cuantitativo de la PCR en tiempo real incluyendo en los experimentos curvas estándar de ARN viral sintético, sería necesario revisar los puntos de normalidad de la prueba en concordancia con las copias de virus en una muestra, especialmente en pacientes con infección asintomática.

El presente estudio tiene varias limitaciones. En primer lugar, el tipo de diseño no permite hacer inferencias acerca de las cadenas de transmisión en cuanto a si la infección ocurrió en el ámbito comunitario o laboral y, aunque ello podría controlarse mediante el estudio de las secuencias genómicas de los pacientes infectados, hasta la fecha dicho análisis no se ha hecho. Por otro lado, están los aspectos relacionados con la capacidad operativa de la RT-PCR para detectar el ARN viral, lo cual se trató de controlar siguiendo el protocolo estandarizado para la toma de las muestras y con el entrenamiento de quienes las tomaron y procesaron.

En resumen, el seguimiento de esta cohorte de trabajadores sanos evidenció la presencia de infección asintomática y leve por SARS-CoV-2 en esta población. Es necesario seguir desarrollando este tipo de estudios para conocer la dinámica de la transmisión de la infección en otros contextos ocupacionales diferentes al de los trabajadores de la salud.
